# Effect of mRNA-LNP components of two globally-marketed COVID-19 vaccines on efficacy and stability

**DOI:** 10.1038/s41541-023-00751-6

**Published:** 2023-10-11

**Authors:** Lizhou Zhang, Kunal R. More, Amrita Ojha, Cody B. Jackson, Brian D. Quinlan, Hao Li, Wenhui He, Michael Farzan, Norbert Pardi, Hyeryun Choe

**Affiliations:** 1 Division of Infectious Disease, Boston Children’s Hospital, Boston, MA USA; 2 Department of Pediatrics, Harvard Medical School, Boston, MA USA; 3 Department of Immunology and Microbiology, UF Scripps Institute for Biomedical Innovation & Technology, Jupiter, FL USA; 4 Skaggs Graduate School, The Scripps Research Institute, La Jolla, CA USA; 5 Center For Integrated Solutions for Infectious Diseases, The Broad Institute of MIT and Harvard, Cambridge, MA USA; 6 Department of Microbiology, Perelman School of Medicine, University of Pennsylvania, Philadelphia, PA USA

**Keywords:** RNA vaccines, SARS-CoV-2, RNA vaccines

## Abstract

During the COVID-19 pandemic, Pfizer-BioNTech and Moderna successfully developed nucleoside-modified mRNA lipid nanoparticle (LNP) vaccines. SARS-CoV-2 spike protein expressed by those vaccines are identical in amino acid sequence, but several key components are distinct. Here, we compared the effect of ionizable lipids, untranslated regions (UTRs), and nucleotide composition of the two vaccines, focusing on mRNA delivery, antibody generation, and long-term stability. We found that the ionizable lipid, SM-102, in Moderna’s vaccine performs better than ALC-0315 in Pfizer-BioNTech’s vaccine for intramuscular delivery of mRNA and antibody production in mice and long-term stability at 4 °C. Moreover, Pfizer-BioNTech’s 5′ UTR and Moderna’s 3′ UTR outperform their counterparts in their contribution to transgene expression in mice. We further found that varying N1-methylpseudouridine content at the wobble position of mRNA has little effect on vaccine efficacy. These findings may contribute to the further improvement of nucleoside-modified mRNA-LNP vaccines and therapeutics.

## Introduction

Following the worldwide spread of COVID-19, rapid implementation of vaccination against SARS-CoV-2 played a critical role in protecting people from serious disease and death. Unlike conventional vaccines such as inactivated virus, protein subunit, DNA, or recombinant viral vector vaccines, the COVID-19 nucleoside-modified mRNA-LNP vaccines from Pfizer-BioNTech (BNT162b2) and Moderna (mRNA-1273), Comirnaty and Spikevax, respectively, were swiftly developed^1,2^. LNP delivery allows the mRNA to enter to the cells and utilize the host cell’s machinery to transiently express antigen in situ. This platform technology exhibits several notable strengths over other modalities. First, native post-translational modification and conformation are similar to the spike protein expressed from natural infection, promoting appropriate humoral and cytotoxic T cell responses^3,4^. Second, the mRNA is non-infectious, non-integrating, and degraded in several days to a few weeks^5^, thus there is no potential risk of infection, insertional mutagenesis, or uncontrolled protein production. Third, as there is no anti-vector immunity against the mRNA, mRNA-LNP vaccines can be administered repeatedly^5^, a notable impediment to the use of viral vectors. As a result, the clinical success of nucleoside-modified mRNA-LNP vaccines surpassed other competing COVID-19 vaccine platforms due to their faster development, better safety, and highest level of protective efficacy^6–8^.

In fact, the concept of mRNA therapeutics including mRNA vaccines has been suggested since mRNA was discovered in 1961^9^, but only recently gained attention as hurdles were overcome, including the instability of in vitro transcribed (IVT) mRNA, inefficient in vivo delivery, and stimulation of undesirable inflammatory responses^10^. For efficient translation, IVT mRNA should have essential structural elements including a 5′ cap, 5′ and 3′ untranslated regions (UTRs) and a poly(A)-tail surrounding the gene of interest. In the past decades, numerous studies have focused on these elements to increase the stability and decrease the immunogenicity of IVT mRNA^5,11^. For example, since the 5′ capping of IVT mRNA, especially the cap1 structure, is critical to facilitate translation initiation and to protect it from degradation by innate immune mechanisms^12^, many approaches have been explored to achieve highly efficient 5′ capping of IVT mRNA^13–15^. In general, the capping of mRNA has been accomplished either by a post-transcriptional enzymatic reaction or a co-transcriptional capping reaction^16,17^. The 5′ cap1 of Moderna’s mRNA was added post-transcriptionally using a vaccinia virus capping enzyme and vaccinia 2′-O-methyltransferase^2^. Although such post-transcriptional enzymatic reaction can achieve 100% capping efficiency^18^, the process is costly and takes longer compared to the co-transcriptional reaction. In contrast, recently developed co-transcriptional trinucleotide cap1 analog (CleanCap) also provides nearly 100% capping efficiency^19^ and is used in the Pfizer-BioNTech mRNA COVID-19 vaccine^1^.

In addition to the mRNA capping strategy, the UTRs of the mRNA are also important to control gene expression. The 5’ UTR, as the entry point for the ribosome during translation, can adopt elaborate RNA secondary and tertiary structures to regulate translation initiation^20^. Highly stable secondary structures within mRNAs can impede ribosome scanning, while structures of lower thermal stability also affect translation if close enough to the 5′ cap^21,22^. Further, appropriate 5′ cap-to-hairpin distance, GC content, and presence of the Kozak sequence must be considered for the design of 5′ UTR^21,23^. Compared to the 5′ UTR, the 3′ UTR regulates the fates and utilities of the mRNAs, including degradation, translation, and localization as it harbors distinct regulatory signals that bind to effectors such as RNA-binding proteins (RBPs), micro RNAs, or other non-coding RNAs^24,25^. For example, AU-rich elements are the known mRNA destabilizing motifs found in 3′ UTRs of many mRNAs^26,27^. In addition to its composition, the length of the 3’UTR also plays a vital role in determining both the stability and the translational efficiency of an mRNA. Those with longer 3′ UTRs have a shorter half-life^28^ whereas mRNAs with shorter 3′UTRs are less efficiently translated^29^. The UTRs at both ends of the spike mRNA of COVID-19 vaccines differ between Pfizer-BioNTech and Moderna. To our knowledge, no study has yet compared the effect of these UTRs on mRNA translation.

Apart from optimizing structural elements of the mRNA, nucleotide modifications through codon optimization or uridine replacement with N1-methylpseudouridine (m1Ψ) can also greatly enhance gene expression. Incorporation of modified nucleosides found in natural RNAs, such as pseudouridine (Ψ), can reduce the TLR-mediated innate immunity against mRNA^30^ and increase the translational capacity and biological stability of the mRNA^31,32^. More recently, it was shown that m1Ψ also naturally found in 18 S rRNA, outperforms Ψ in mRNA modification by providing enhanced protein expression and reducing innate immunity against mRNA in mammalian cell lines and mice^33^. Therefore, considering the beneficial effect of m1Ψ in mRNA immunogenicity and stability, both Pfizer-BioNTech (BNT162b2) and Moderna (mRNA-1273) have replaced all the uridines of the mRNA with m1Ψ during in vitro transcription^1,2^. However, both have different m1Ψ content due to their different codon optimization strategies, and it is currently unknown whether different m1Ψ content of these vaccines plays a role in mRNA stability.

**Fig. 1:**
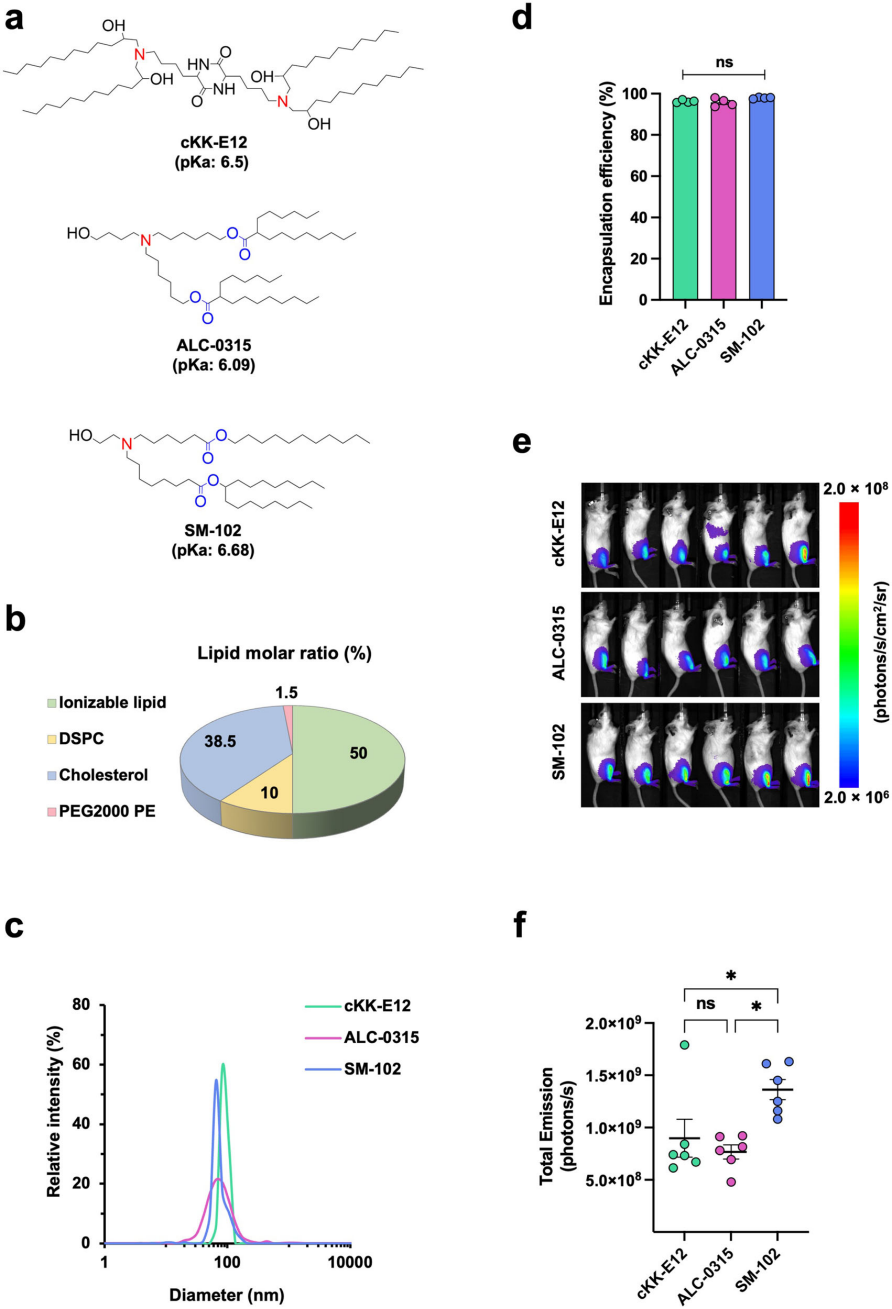
SM-102 ionizable lipid is moderately more efficient than ALC-0315 in intramuscular delivery of mRNA in mice. **a** Chemical structures of ionizable lipids cKK-E12, ALC-0315, and SM-102. Protonatable nitrogens are indicated in red, and biodegradable ester bonds in blue. Shown pKa values are apparent pKa. **b** A pie chart showing the molar ratio of lipids for LNP formulation. DSPC: Distearoylphosphatidylcholine; PEG2000 PE: 1,2-dioleoyl-sn-glycero-3-phosphoethanolamine-N-[methoxy(polyethylene glycol)-2000]. **c** Size distribution of Fluc mRNA-LNPs was measured by dynamic light scattering (DLS) and plotted with particle size on the X axis and relative intensity of scattered light on the Y axis. Plots shown are representative of two independent experiments, each conducted with two independent Fluc mRNA-LNP preparations. **d** Encapsulation efficiency of Fluc mRNA-LNPs was determined by Quant-iT™ RiboGreen RNA Assay Kit. Encapsulation Efficiency (%) = [(Fluorescence)_total mRNA_ – (Fluorescence)_outside mRNA_] / (Fluorescence)_total mRNA_ × 100%. **e** Images of mice at 24 h after injected with 1 μg Fluc mRNA-LNP. Bioluminescence intensity is presented in radiance (photons/s/cm2/sr) in a rainbow scale. **f** Bioluminescence shown in **e** was quantified as total emission in the region of interest. Each dot represents an individual mouse. Two independent preparations of mRNA-LNPs were used for each group. Data are presented as Mean ± SEM of *n* = 6 mice per group. Statistical significance among the groups was analyzed by one-way ANOVA with Tukey’s multiple comparisons test (ns, not significant; *p < 0.05).

In parallel with the progress in mRNA research, the development and optimization of the lipid nanoparticle (LNP) delivery system^34–37^ has facilitated rapid growth in the field of mRNA therapeutics. In particular, LNPs formulated with ionizable lipids have gained attention because they are positively charged at acidic pH, necessary to bind to and package RNAs into LNPs, but are neutral at physiological pH to minimize toxicity. As a result, LNPs formulated with ionizable lipids have fewer interactions with the anionic membranes of blood cells when they are neutral at physiological pH and, thus, improve the biocompatibility for mRNA delivery in vivo^34,38,39^. Further, they are protonated in the acidic endosome after cellular uptake and thus interact with anionic endosomal phospholipids to form cone-shaped ion pairs that facilitate membrane fusion and disruption, endosomal escape, and cargo release into the cytosol^40,41^. While both ALC-0315 ionizable lipid employed by Pfizer-BioNTech and SM-102 by Moderna look chemically similar (Fig. 1a), it is unknown whether their mRNA delivery efficiency is also comparable in vivo.

Despite the abundant data in efficacy of the Pfizer-BioNTech and Moderna vaccines in humans, there is no detailed comparison so far in their specific components except one in silico analysis^42^. In this study, we sought to dissect and compare several key components of these vaccines, including the ionizable lipids, UTRs, and mRNA modification, for their performance in gene expression and ability to facilitate antibody production. We found that SM-102 ionizable lipid outperforms ALC-0315 in intramuscular delivery of mRNA, displayed by higher luciferase expression, as well as LNP stability in vitro when stored at 4 °C. We also found that the 5′ UTR of Pfizer-BioNTech mRNA is more efficient than that of Moderna’s in mRNA translation, while the opposite is true for the 3′ UTR. In contrast, we observed that the m1Ψ content difference in mRNA at the wobble positions has little effect on neutralizing antibody production. These findings could provide valuable information for improving mRNA vaccines and therapeutics.

## Results

### SM-102 ionizable lipid is moderately more efficient than ALC-0315 in intramuscular delivery of mRNA in mice

Given the key role of the ionizable lipid in LNP mediated mRNA delivery, we asked how mRNA intramuscular delivery efficiency was affected by ALC-0315 (Pfizer-BioNTech) versus SM-102 (Moderna) ionizable lipids. To do so, firefly luciferase (Fluc) mRNA-LNPs were formulated with either ALC-0315 or SM-102 together with the same helper lipids as shown in Fig. 1b. Another mRNA-LNP containing cKK-E12^43,44^, a commonly studied ionizable lipid, was included for a parallel comparison. These three Fluc mRNA-LNPs were first characterized by particle size and mRNA encapsulation efficiency. The results showed that when mixed with cholesterol, DSPC, and PEG2000 PE at a ratio indicated in Fig. 1b, SM-102 LNP has slightly smaller size (75.5 ± 0.4 nm) compared to the ALC-0315 LNP (90.2 ± 7.8 nm) and cKK-E12 LNP (88.2 ± 1.5 nm), and that ALC-0315 LNP forms a slightly wider size distribution (Fig. 1c). However, they all have very similar encapsulation efficiency of over 95% (Fig. 1d). Next, we evaluated their mRNA delivery efficiency by injecting 1 μg of Fluc mRNA-LNP into the gastrocnemius muscle of BALB/c mice. Using in vivo bioluminescent imaging at 24 h post-injection, we observed that cKK-E12 and ALC-0315 achieved similar luciferase protein expression while mean bioluminescence mediated by SM-102 was 60% higher (Fig. 1e, f). Taken together, we found that in our experimental conditions, SM-102 is the best ionizable lipid among these three for efficient intramuscular mRNA delivery that leads to high protein expression in mice.

### SM-102 elicits comparable inflammatory response but higher antibody production in mice compared to ALC-0315

**Fig. 2:**
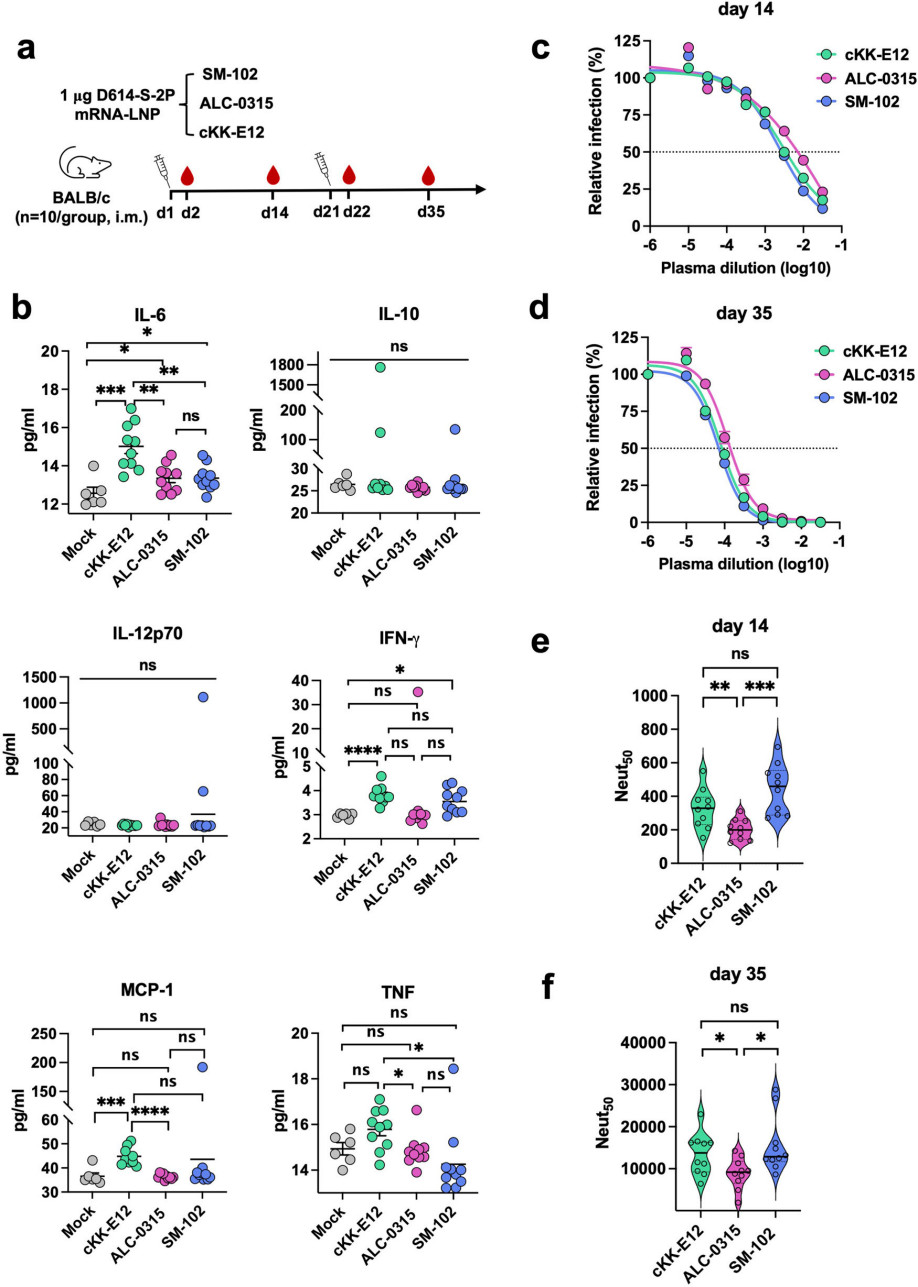
SM-102 elicits comparable inflammatory response but higher antibody production in mice compared to ALC-0315. **a** Timeline of mouse immunization and bleeding. Ten BALB/c mice per group were intramuscularly vaccinated at the indicated days, each time with 1 μg SARS-CoV-2 spike mRNA-LNP prepared with the indicated ionizable lipid. Blood was collected at the indicated time points to measure cytokines and neutralizing antibodies. **b** Pro-inflammatory cytokines/chemokines in the plasma collected at day 2 were detected using the murine inflammation kit and analyzed by Accuri Flow cytometer. Data are presented as Mean ± SEM of *n* = 10 mice per group. Cytokine levels in the d22 plasma are presented in Supplementary Fig. 1. **c**, **d** SARS-CoV-2 pseudovirus (PV) was preincubated with or without (presented at *x* = − 6) serially diluted plasma collected at day 14 **c** or 35 **d**. H1299-hACE2 cells were incubated with these preincubated mixes and analyzed 24 h later by measuring luciferase activity. Entry of SARS-CoV-2 PV in the presence of immune plasma relative to that in the absence of plasma is shown. Each dot on the curve represents the average value from ten mice. The dashed lines in the figures indicate 50% neutralization. **e**, **f** Violin plots show the Neut_50_ value of individual mouse plasmas (*n* = 10 per group) collected at day 14 **e** or 35 **f**. The central thick lines indicate the median of ten Neut_50_ values per group. Statistical significance among the groups was analyzed by one-way ANOVA with Tukey’s multiple comparisons test (ns, not significant; *p < 0.05, **p < 0.01, ***p < 0.001, and ****p < 0.0001).

Considering that protective immunity requires not only robust expression of the transgene, but also strong stimulation of antibody production, we next evaluated the effect of ALC-0315 and SM-102 on antibody production. LNPs were formulated with SARS-CoV-2 spike mRNA, which encodes a full-length spike protein identical to that of Pfizer-BioNTech and Moderna mRNA vaccines. This spike protein has aspartic acid at the residue 164 and is stabilized by the diproline mutation, K986P and V987P (D614-S-2P). cKK-E12 ionizable lipid was again included as a control. Ten BABL/c mice per group were primed and boosted with 1 μg of indicated mRNA-LNP by intramuscular (i.m.) injection at day 1 and day 21, respectively, as depicted in Fig. 2a. Twenty-four hours post first and second injections, mice plasmas were collected to assess inflammatory response and analyzed by cytokine bead array (CBA). In general, there was no significant inflammatory response induced by ALC-0315 or SM-102 LNP compared to mock (pre-immune plasmas) in either d2 or d22 plasmas (Fig. 2b, Supplementary Fig. 1). In contrast, cKK-E12 LNP triggered stronger inflammatory cytokine production, especially for IL-6, IFN-γ, and MCP-1 at d2, which is not observed at d22 except MCP-1. At day 14 and day 35, mice plasmas were collected again for detection of neutralizing antibodies (NAbs) against SARS-CoV-2 spike. Days 14 and 35 were selected to detect the difference at an early stage of immune reaction, when a biggest difference can be observed, and after antibody production reaches near maximum at two weeks after a boost vaccination when a smallest difference is expected, respectively. Serially-diluted plasmas were incubated with retroviral pseudovirus (PV) bearing the spike protein on H1299 human lung epithelial cells exogenously expressing hACE2. In our experimental conditions where all other components are the same, SM-102 and cKK-E12 LNP elicited small but significantly higher neutralizing activity than did ALC-0315 LNP (Fig. 2c, e) at day 14. Similarly, SM-102 and cKK-E12 LNP groups maintained higher neutralization titer than that of ALC-0315 LNP group post-boost at day 35 (Fig. 2d, f) although the antibody titer was increased >28-fold in all groups after the boost.

### Sucrose enhances intramuscular delivery of SM-102 and ALC-0315 mRNA-LNP in mice

**Fig. 3:**
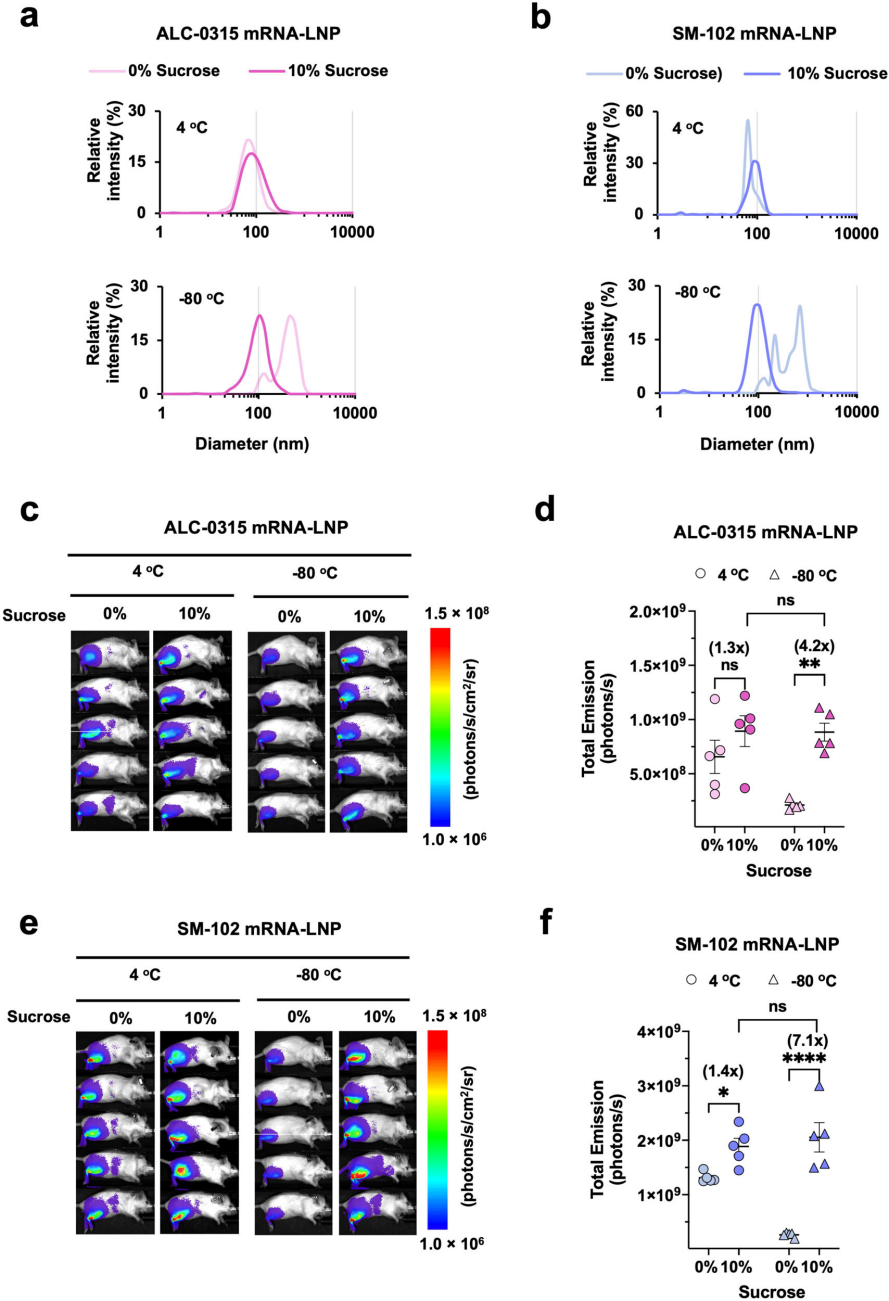
Sucrose enhances intramuscular delivery efficiency of SM-102 and ALC-0315 mRNA-LNP in mice. **a**, **b** The particle size distribution of ALC-0315 **a** or SM-102 **b** Fluc mRNA-LNP stored for 1 h at 4 °C (upper panel) or −80 °C (lower panel) with or without 10% sucrose was measured by DLS. Particle size is plotted on the X axis with relative intensity of scattered light on the Y axis. Plots shown are representative of two independent experiments, each conducted with two independent Fluc mRNA-LNP preparations. **c**, **e** Images of mice at 24 h post intramuscular injection of 1 μg Fluc mRNA-LNP formulated with either ALC-0315 **c** or SM-102 **e**. Bioluminescence intensity is shown in radiance (photons/s/cm2/sr) in a rainbow scale. **d**, **f** Bioluminescence imaged in **c**, **e**, respectively, was quantified as total emission in the region of interest. Each dot represents an individual mouse. Data are presented as Mean ± SEM of *n* = 5 mice per group. Statistical significance among the groups was analyzed by two-way ANOVA with Sidak’s multiple comparisons test (ns, not significant; *p < 0.05, **p < 0.01, and ****p < 0.0001).

Sucrose as a cryoprotectant is a key component of the COVID-19 mRNA-LNP vaccines for their ultracold chain transportation. However, it is unclear whether sucrose would have any effect other than cryoprotectant on mRNA-LNP vaccine. To evaluate this point, ALC-0315 and SM-102 LNP encapsulating Fluc mRNA were prepared in the absence or presence of 10% sucrose and then stored at 4 °C or snap frozen at −80 °C for 1 h. Before administering these Fluc-mRNA-LNPs to mice, their particle sizes and encapsulation efficiencies were measured. For the LNPs stored at 4 °C, we noticed that 10% sucrose modestly increased the particle size of both ALC-0315 LNP (83.8 ± 4.6 nm in PBS vs 100.1 ± 5.2 nm in PBS + 10% sucrose) and SM-102 LNP (74.4 ± 5.1 nm in PBS vs 89.7 ± 5.6 nm in PBS + 10% sucrose) compared to those without sucrose (Fig. 3a, b, upper panels). We tested varying sucrose concentrations on LNP size and observed a dose effect between 2 and 10% but not at above 10% (Supplementary Fig. 2). As expected, sucrose protected both ALC-0315 and SM-102 Fluc mRNA-LNP from aggregation during freezing-thawing as shown by the clean single peak compared to the multiple peaks and wide size distribution of the LNPs without sucrose (Fig. 3a, b, lower panels). After intramuscular administration into mice of 20 μl LNPs (approximately 1 μg mRNA) stored at 4 °C, we found that 10% sucrose enhanced luciferase expression by 30–40% for both ALC-0315 and SM-102 LNP (Fig. 3c–f). For −80 °C frozen LNPs, mRNA delivery efficiency in vivo was fully preserved as expected when sucrose is present in LNP during freezing-thawing (Fig. 3c–f). Hence, these data confirm sucrose is a cryoprotectant and also suggest that it could increase intramuscular delivery of mRNA when LNPs are stored at 4 °C.

### ALC-0315 mRNA-LNP is moderately less stable than SM-102 mRNA-LNP at 4 °C

**Fig. 4:**
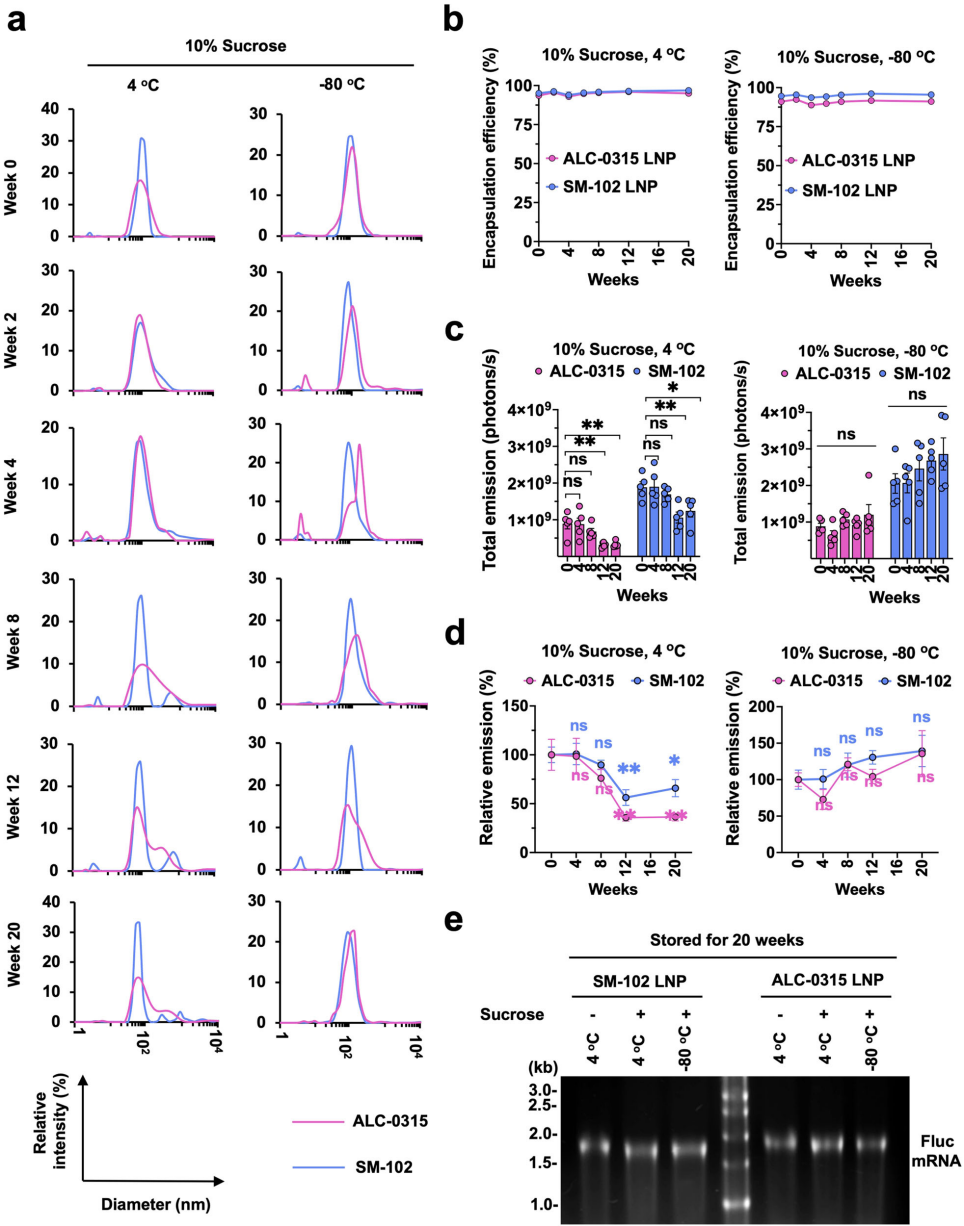
ALC-0315 mRNA-LNP is moderately less stable than SM-102 mRNA-LNP at 4 °C. **a** Particle size of ALC-0315 or SM-102 Fluc mRNA-LNP stored at 4 °C and −80 °C was measured by DLS at indicated time points. Particle size is plotted on the X axis with relative intensity of scattered light on the Y axis. Plots shown are representative of two independent experiments, each conducted with two independent Fluc mRNA-LNP preparations. **b** Encapsulation efficiency of the Fluc mRNA-LNP shown in **a** was measured at the indicated time points, using Quant-iT^TM^ RiboGreen RNA Reagent. **c** Bioluminescence from the mice was quantified as total emission in the region of interest at 24 h after the injection of the Fluc mRNA-LNP at the indicated time points. **d** Bioluminescence values shown in **c** were normalized to that measured at week 0. Data are presented as Mean ± SEM of *n* = 5 mice per group. Statistical significance between week 0 and other time points within the same group was analyzed by one-way ANOVA with Dunnett’s multiple comparisons test. (ns, not significant; *p < 0.05 and **p < 0.01). **e** Encapsulated mRNA in LNP after 20 weeks of storage at 4 °C or −80 °C. 4 μl of 50 μg/ml mRNA-LNP was loaded per lane. Image shown is the representative of two independent mRNA-LNP preparations.

To overcome the challenges of ultracold transportation, particularly for distributing vaccines to remote areas, people have explored methods like lyophilization to store mRNA-LNP at ambient temperature^45^. However, because the ionizable lipid they tested is not the same as those used by Pfizer-BioNTech or Moderna, it is unclear whether ultracold storage is indispensable for the transportation of these two vaccines or how stable they would be at different storage conditions. Therefore, we investigated the stability of SM-102 and ALC-0315 LNP encapsulating nucleoside-modified Fluc mRNA at 4 °C and −80 °C for up to 20 weeks. Every two weeks during the 20 weeks, size, and encapsulation efficiency of the mRNA-LNP were measured, and on the same day, these mRNA-LNPs were intramuscularly administered into mice. We found that both ALC-0315 and SM-102 mRNA-LNPs started to aggregate in the presence of 10% sucrose at 8 weeks of storage at 4 °C but not at −80 °C (Fig. 4a), although the encapsulation efficiency was stable for all storage conditions at all time points (Fig. 4b). We also observed that the luciferase expression in vivo gradually decreased after 4–8 weeks if mRNA-LNPs were stored at 4 °C but did not decrease if stored at −80 °C (Fig. 4c). Decreasing luciferase expression appears to correlate with the aggregation status of the LNPs (Fig. 4a, c). When we analyzed the data shown in Fig. 4c for their relative values, we noticed that at 4 °C, the ALC-0315 mRNA-LNP lost its stability faster than SM-102 mRNA-LNP, while both were stable or even moderately increased with time at −80 °C (Fig. 4d). To investigate whether the decreased luciferase expression in vivo might result from Fluc mRNA degradation at 4 °C, the mRNA-LNPs stored at 4 °C or −80 °C for 20 weeks were lysed, and their mRNA was visualized by gel electrophoresis and ethidium bromide staining. Figure 4e shows encapsulated mRNA was stable regardless of storage temperature. Taken together, SM-102 and ALC-0315 LNPs encapsulating Fluc mRNA are stable only for short-term at 4 °C with SM-102 LNP being more stable than ALC-0315 LNP, but both are stable for a long-term storage at −80 °C.

### UTRs from Moderna and Pfizer-BioNTech mRNA differentially contribute to translation

**Fig. 5:**
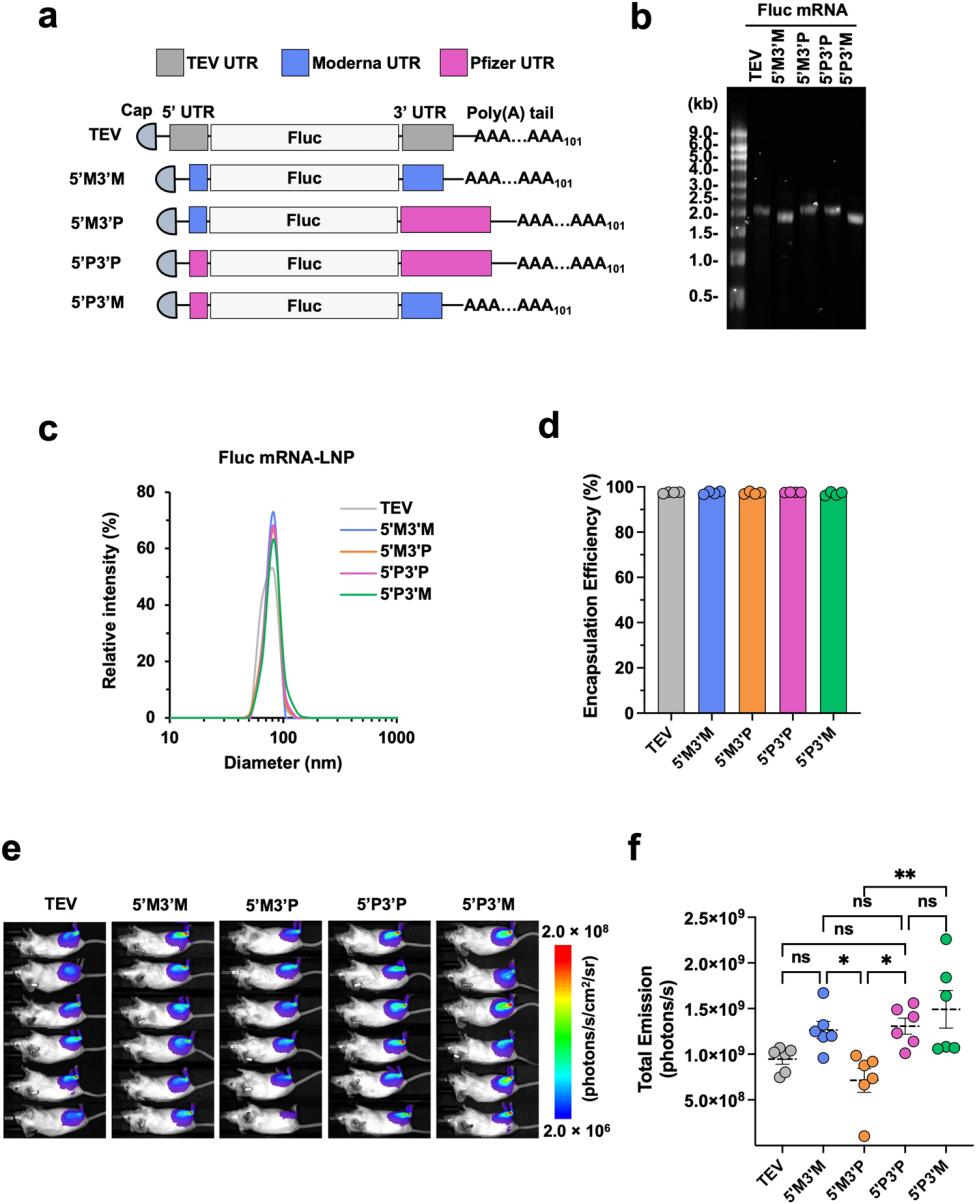
UTRs from Moderna and Pfizer-BioNTech mRNA differentially contribute to translation. **a** Schematic diagrams of the Fluc mRNAs with different UTRs but with the same 5’ cap and 3’ poly(A) tail of 101 nucleotides. **b** Size and purity of Fluc mRNAs were checked by RNA gel electrophoresis. **c** Size distribution of the LNPs shown in **b** was measured by DLS. Particle size is plotted on X axis and relative intensity of scattered light on Y axis. Presented plots are the representative of two independent experiments, each conducted with two independent Fluc mRNA-LNP preparations. **d** Encapsulation efficiency of the same Fluc mRNA-LNPs. **e** Bioluminescence images of mice at 24 h post intramuscular injection of 1 μg Fluc mRNA-LNP containing the indicated UTRs. Bioluminescence intensity is presented in radiance (photons/s/cm2/sr) in a rainbow scale. **f** Bioluminescence shown in **e** was quantified as total emission in the region of interest. Each dot represents an individual mouse. Data are presented as Mean ± SEM of *n* = 6 mice per group. Statistical significance among the groups was analyzed by one-way ANOVA with Tukey’s multiple comparisons test (ns, not significant; *p < 0.05 and **p < 0.01).

The 5′ and 3′ UTRs of the spike mRNA in Moderna and Pfizer-BioNTech COVID-19 vaccines are different in their sequence and structure (Supplementary Fig. 3). To understand whether this difference impacts mRNA translation, we produced Fluc mRNA containing either Moderna’s UTRs (5′M3′M) or Pfizer-BioNTech’s UTRs (5′P3′P), or the UTRs of another mRNA vector (TEV) (Fig. 5a). In order to compare the effect of 5′ and 3′ UTR separately, we designed additional Fluc mRNAs (5’M3’P and 5’P3’M) by swapping one side of the UTRs between 5′M3′M and 5’P3’P (Fig. 5a). All these five Fluc mRNAs were in vitro transcribed in the presence of m1Ψ and displayed comparable purity after cellulose column purification (Fig. 5b). Next, we generated LNPs with these five Fluc mRNAs with identical cKK-E12 and other lipid components. These LNPs exhibit comparable particle size and encapsulation efficiency (Fig. 5c, d). Mice were intramuscularly injected with 1 μg of these Fluc mRNA-LNPs and imaged at 24 h post-injection, and we found there was no significant difference among TEV, 5′M3′M and 5′P3′P (Fig. 5e, f). However, when we focused on the effect of individual UTR, we found that 5′P3′P expressed higher Fluc than that of 5’M3’P, suggesting Pfizer-BioNTech’s 5′ UTR promotes higher expression than does Moderna’s 5′ UTR. In contrast, Pfizer-BioNTech’s 3′ UTR leads to lower expression than Moderna’s 3′ UTR as 5′M3′P expressed lower Fluc than did 5′M3′M. Therefore, the highest Fluc expression was achieved by 5′P3′M (Fig. 5e, f). Overall, our data show that the original UTRs from Pfizer-BioNTech and Moderna mRNA work comparably and indicate that individual UTR contributes differently to mRNA translation with Pfizer-BioNTech’s 5′ UTR and Moderna’s 3’ UTR being modestly more efficient than their counterparts.

### m1Ψ content at the wobble position of RBD mRNA does not significantly contribute to vaccine efficacy in mice

**Fig. 6:**
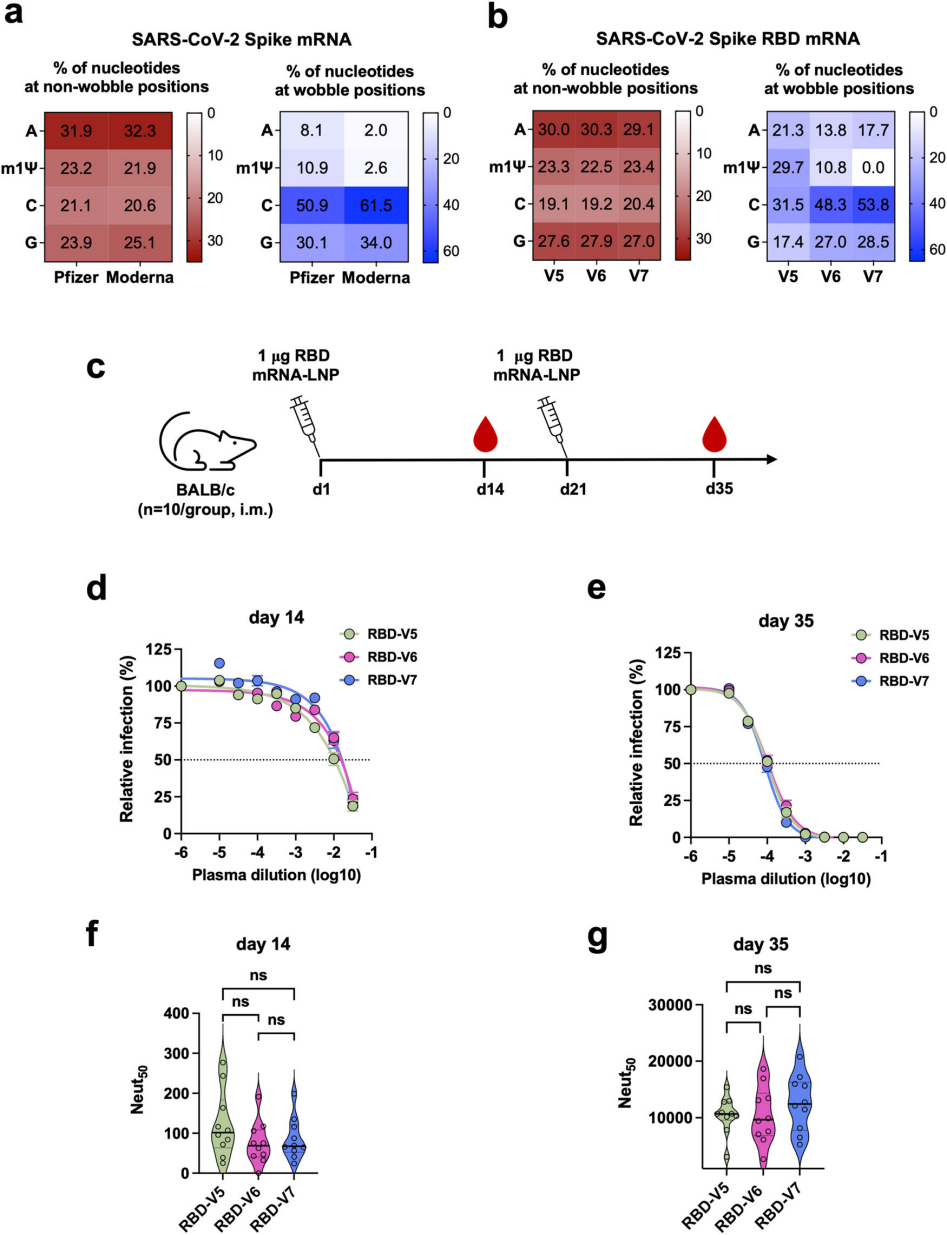
m1Ψ content at the wobble position of RBD mRNA does not significantly contribute to vaccine efficacy in mice. **a** Nucleotide composition of the spike mRNA of Pfizer-BioNTech and Moderna COVID-19 vaccines. The numbers shown in the boxes are the percentage of the indicated nucleotide in those at non-wobble positions (left panel) or at the wobble position (right panel). **b** Nucleotide composition of three spike RBD mRNAs designed to contain varying level of wobble m1Ψ content. RBD-V6 and RBD-V7 mRNA have wobble m1Ψ content close to that of the Pfizer-BioNTech and Moderna COVID-19 vaccines, respectively. Otherwise, these RBD mRNAs encode the identical protein and share the same 5′ cap, UTRs, and poly(A) tail. Their LNPs were formulated with the same lipids. **c** Timeline of mouse immunization and bleeding. **d**, **e** SARS-CoV-2 PV neutralization assays conducted with plasmas collected at days 14 **d** and 35 **e**. Each dot on the curves represents the average value from ten mouse plasmas. Dashed lines indicate 50% neutralization. **f**, **g** Violin plots show the Neut_50_ values of mouse immune plasmas (*n* = 10 per group) shown in **d**, **e**, respectively. Each dot represents an individual mouse. The thick horizontal lines and error bars indicate Mean ± SEM of *n* = 10 mice per group. Statistical significance among the groups was analyzed by one-way ANOVA with Tukey’s multiple comparisons test (ns, not significant).

Whereas both Pfizer-BioNTech and Moderna COVID-19 vaccines encode the identical spike protein of SARS-CoV-2, their nucleotide sequences, especially at the wobble position, are substantially different due to the different codon optimization strategies used (Fig. 6a). Moderna has replaced most of the adenine and thymine at wobble position with guanine and cytosine, which leads to 14.5% higher GC content at the wobble position of the spike gene than that of Pfizer-BioNTech. Although high GC content might be beneficial for mRNA translation^46,47^ or stabilization^48^, it reduces the content of uridine in mRNA that can be replaced by m1Ψ during in vitro transcription. M1Ψ enhances vaccine efficacy by stabilizing mRNA and facilitating evasion from host innate immunity^30,31^. To explore whether m1Ψ content, especially that at the wobble position (wobble m1Ψ), influences vaccine efficacy, three mRNAs encoding the identical Receptor Binding Domain (RBD) of SARS-CoV-2 spike but containing varying levels of wobble m1Ψ were generated (Fig. 6b). Briefly, RBD-V5 mRNA contains the highest wobble m1Ψ (29.7%), while RBD-V6 (10.8%) and -V7 (0%) mRNAs contain wobble m1Ψ content close to that of Pfizer-BioNTech (10.9%) and Moderna (2.6%) spike mRNA, respectively. To evaluate only the effect of wobble m1Ψ content, these RBD mRNAs were generated with the same UTRs and a poly(A) tail (Supplementary Fig. 4a) derived from the TEV vector, and LNPs were formulated with these RBD mRNAs and the same cKK-E12 and other helper lipids. All three mRNA-LNPs exhibited comparable encapsulation efficiency and particle size (Supplementary Fig. 4b, c). 1 μg of these RBD mRNA-LNPs were intramuscularly injected per mouse on days 1 and 21, and plasmas were collected on days 14 and 35 (Fig. 6c). Neutralization assays conducted using SARS-CoV-2 PV showed no significant difference in NAbs titers in the plasmas collected at both days 14 and 35 post vaccination with these three RBD mRNA-LNPs (Fig. 6d–g). These data show that in the case of RBD mRNA, the wobble m1Ψ content between 0% (similar to Moderna’s) and 10.8% (similar to Pfizer-BioNTech’s) does not result in significant difference in mRNA vaccine efficacy in mice.

## Discussion

Ionizable lipids, the key component of the LNP, account for approximately 50% of the total lipids and play a vital role in mRNA delivery in mRNA-LNP vaccines. Here, we compared the efficiency of two ionizable lipids ALC-0315 (Pfizer-BioNTech) and SM-102 (Moderna) in mRNA delivery. To investigate only the ionizable lipids, other LNP components were kept the same, and thus the compositions of these LNPs are not the same as those of the Pfizer-BioNTech or Moderna vaccines. In this condition, we found that SM-102 outperforms ALC-0315 in mice when administered intramuscularly (Fig. 1). One contributing factor for different delivery efficiency is their endosomal escape ability determined by the pKa values and chemical structures of the ionizable lipids (Fig. 1a). Once LNPs are internalized by the cell, the charge of ionizable lipids increases as pH decreases below its pKa in the endosome (pH 7.0 to 5.5), which brings about two effects. First, osmotic swelling of the endosome, as the accumulation of protons with counter ions enhances the transportation of liquids from the cytosol to the endosome to counteract the osmotic pressure^49^. Second, ion pair formation between cationic ionizable lipids with anionic endosomal phospholipids^50^. In the meantime, as cone-shaped ionizable lipids with a small head group and a broader tail (see chemical structure of SM-102 and ALC-0315 in Fig. 1a) are incompatible with cylindrical lipids in the lipid bilayer of the endosome, the endosomal membrane is destabilized, and mRNA is released into the cytosol for subsequent translation. Theoretically, the higher the pKa of an ionizable lipid to endosomal pH, the greater the ability to accept protons, thereby leading to more effective endosome escape. This explains, at least partially, why Fluc expression mediated by SM-102 (pKa = 6.68)^51^ was more efficient than that mediated by ALC-0315 (pKa = 6.09)^52^ or cKK-E12 (pKa = 6.5)^53^, as our study shows (Fig. 1e, f).

Given that luciferase expression in mice only reflects mRNA-LNP delivery efficiency but does not predict vaccine efficacy because it is also contributed by the intrinsic adjuvant activity of various LNPs^54–57^, we compared the induction of inflammatory cytokines and chemokines as well as the NAb production after mice were immunized with cKK-E12, ALC-0315, or SM-102 LNPs encapsulating the same SARS-CoV-2 spike mRNA (Fig. 2). SM-102 and ALC-0315 mRNA-LNPs induced comparable level of cytokines and chemokines, while cKK-E12 mRNA-LNP elicited stronger inflammatory responses than the other two particularly with higher IL-6, IFN-γ, and MCP-1 production (Fig. 2b). Multiple previous studies demonstrated that IL-6 and IFN-γ directly or indirectly stimulated antibody production by promoting B cell growth or maturation^57–62^. Consistently, higher NAb titer was observed in the cKK-E12 mRNA-LNP group (Fig. 2e, f) compared to the ALC-0315 mRNA-LNP group despite their comparable transgene expression levels (Fig. 1f). Although the mechanism for why cKK-E12 induces stronger inflammatory responses is unclear, one possible explanation is that cKK-E12 is degraded more slowly than SM-102 and ALC-0315, thereby resulting in excess fatty acid accumulation, which contributes to inflammation and cytokine release^63,64^. As a matter of fact, cKK-E12 doesn’t have the biodegradable ester bonds that are stable at physiological pH but enzymatically hydrolyzed within tissues and cells^40^, whereas SM-102 and ALC-0315 do (Fig. 1a).

In addition, we examined whether ALC-0315 and SM-102 differentially contribute to mRNA-LNP stability. Fluc mRNA-LNP made with either ALC-0315 or SM-102 but with the same helper lipids was stored at 4 °C or −80 °C and injected into mice at weeks 0, 4, 8, 12, and 20. For −80 °C storage, it is established that sucrose is required to protect LNP from fusion and aggregation during freezing and thawing and thereby retain mRNA-LNP potency^65^, which is confirmed in our study as shown in Fig. 3. However, it was not previously known how sucrose may affect the mRNA-LNP delivery and stability in non-freezing conditions, and thus we investigated the delivery efficiency of mRNA-LNP stored at 4 °C with or without 10% sucrose. In general, sucrose slightly enhanced mRNA-LNP delivery in vivo when injected intramuscularly (Fig. 3c–f). However, sucrose did not contribute to the long-term stability of ALC-0315 or SM-102 mRNA-LNP if stored at 4 °C, as shown by their decreasing potency with time (Fig. 4c). Whereas comparable amount of mRNA was detected in the LNPs stored at both 4 °C and −80 °C for 20 weeks (Fig. 4e), LNP aggregation occurred at 4 °C starting at week 8 or 12 (Fig. 4a), which might have affected LNP uptake resulting in reduced Fluc expression in vivo (Fig. 4c). ALC-0315 LNP showed higher tendency of aggregation than did SM-102 LNP after storage at 4 °C for 8 weeks or longer (Fig. 4a) and exhibited larger loss of potency in vivo (Fig. 4d), suggesting negative effect of LNP aggregation on vaccine efficacy. Another potential mechanism for loss of LNP potency with time is RNA adduct formation that will lead to a degradation pathway, once LNP is delivered to the target cells. More studies are warranted on this subject. Note that these results may not be extendable to the stability of the Moderna and Pfizer-BioNTech vaccines because these data were obtained from controlled studies for apples-to-apples comparisons, in which other components and the manufacturing process are likely different from those of the marketed vaccines.

Aside from long-term stability of mRNA-LNP in vitro, stability and translation capacity of mRNA in vivo is another key contributing factor to the efficacy of mRNA vaccine. Manufacturers have tried their own approaches to achieve this goal, and their strategies are generally quite similar including the optimization of 5′ cap, length of poly-A tail, the composition of untranslated regions (UTRs), codon optimization as well as nucleoside modifications to achieve incorporation of m1Ψ. Given that Pfizer-BioNTech and Moderna spike mRNAs are quite different in the UTR sequences and the wobble m1Ψ content, which results from their distinct codon optimization strategies, our study here focused on the comparison of the effect of these two components on mRNA translation and antibody production (Figs. 5 and 6). Our data show that the UTRs used by these two companies display comparable effect on mRNA translation (Fig. 5e, f). However, when the 5′ and 3′ UTRs were investigated separately, we found that Pfizer-BioNTech 5′ UTR yielded modestly higher luciferase expression than Moderna’s 5′ UTR and that the opposite was true for the 3′ UTRs. Pfizer-BioNTech’s 5′ UTR sequence is derived from the human hemoglobin α-globin (HBA1) gene, an efficient expressor, while the source of Moderna’s 5′ UTR is unclear. Moderna’s 5’ UTR contains a GC-rich tract just upstream of the Kozak sequence^66^, and this GC-rich tract and its secondary structure may reduce translation initiation efficiency and overall protein output^67,68^. For the 3′ UTR, the Pfizer-BioNTech vaccine combines one segment from a human mRNA encoding amino-terminal enhancer of split (AES) and another from mitochondrial 12 S rRNA (mtRNR1)^69^, while 3′ UTR of the Moderna vaccine originates from α-globin (HBA1) gene^42,66^. The Pfizer-BioNTech 3′ UTR (AES-mtRNR1) was previously shown to outperform the two head-to-tail human β-globin (2hBg) 3′ UTR in mRNA stability and protein output^69^. In our study here, Pfizer-BioNTech 3′ UTR was not more efficient than HBA1 3′ UTR in the Moderna vaccine. Besides the fact that they are two distinct genes, the varying results from 2hBg 3′ UTR and HBA1 3′UTR may also reflect that mRNA stability is dependent on specific cell or tissue types^70^. Namely, 2hBg 3′ UTR was compared to AES-mtRNR1 in the lymphatic compartments in a previous study^69^, while we compared HBA1 3′ UTR to AES-mtRNR1 in the muscle tissues (Fig. 5e, f).

Because of the different codon optimization strategies used, Moderna’s spike mRNA has higher GC content than that of Pfizer-BioNTech (Fig. 6a). In general, higher GC content correlates with higher protein expression in mammalian cells due to the codon bias^46,71^ and enhanced mRNA stability^47,48^. In regular mRNA, higher GC content proportionally lowers AU content, thereby increasing mRNA stability. However, in nucleoside-modified mRNA, m1Ψ that replaces uridine can also promote mRNA stability via increased base pairing and stacking^67,72,73^. It is unclear whether the m1Ψ content and GC content compensate each other on mRNA stability and antibody production. Our study shows m1Ψ content at the wobble position does not make a significant difference in antibody titer (Fig. 6). This result indicates that various codon-optimization strategies used for vaccine mRNA may not make a significant difference in antibody generation likely because there is a reciprocal relationship between GC content and m1Ψ level.

In summary, we dissected and compared the effect of ionizable lipid, UTRs, and m1Ψ levels of the mRNA used in the Pfizer-BioNTech and Moderna vaccines on mRNA delivery, protein expression, antibody generation, and long-term mRNA-LNP stability. We found in our experimental conditions the Moderna’s ionizable lipid SM-102 outperforms Pfizer-BioNTech’s ALC-0315 in mRNA delivery, antibody induction in mice, as well as long-term stability at 4 °C but not at −80 °C. We also found the instability of both SM-102 and ALC-0315 LNPs during a long-term storage at 4 °C. We further observed Pfizer-BioNTech’s 5′ UTR and Moderna’s 3′ UTR outperforms their counterparts in mRNA translation, and varying m1Ψ content at the wobble position, when assessed using RBD mRNA, has little effect on vaccine efficacy in mice.

As the extraordinary success of Pfizer-BioNTech and Moderna’s SARS-CoV-2 vaccines affirm, nucleoside-modified mRNA-LNP vaccines will continue to be an important means to address various human diseases. A careful investigation of the components of these vaccines can enhance their efficacy.

## Materials and methods

### Ethics

All study procedures were approved by the Institutional Biosafety Committee, and Institutional Animal Care and Use Committee at The UF Scripps Institute for Biomedical Innovation & Technology. All experiments conform to all relevant regulatory standards.

### Cell lines

HEK293T (human embryonic kidney; ATCC) were maintained in high-glucose DMEM (Cat# 10569-010, Life Technologies) supplemented with 100 U/mL Penicillin and 100 μg/mL Streptomycin (Cat# 15140-122, Life Technologies), and 10% FBS (Cat# 12303C, Sigma-Aldrich) at 37 °C in 5% CO_2_. NCI-H1299 (a lung epithelial cell line) kindly provided by Joseph Kissil (The Scripps Research Institute, Jupiter, FL, USA) was transduced by MLV retrovirus to stably express human ACE2 (hACE2). H1299-hACE2 cells were selected and maintained in RPMI supplemented with 1 μg/ml puromycin and 10% FBS and Penicillin-Streptomycin.

### Lipids

Ionizable lipids SM-102 (Cat# HY-134541) and ALC-0135 (Cat# HY-138170) were purchased from MedChemExpress (MCE) and cKK-E12 (Cat# O-8744) from Organix lnc.. 1,2-distearoyl-sn-glycero-3-phosphocholine (DSPC, Cat# 850365P) and 1,2-dimyristoyl-sn-glycero-3-phosphoethanolamine-N-[methoxy(polyethylene glycol)-2000] (14:0 PEG2000 PE, Cat# 880150P) were obtained from Avanti Polar Lipids. Cholesterol (Cat# C8667) was purchased from Sigma-Aldrich. All lipids were dissolved in absolute ethanol (Cat# BP2818, Fisher Scientific).

### DNA construction

The pUC-ccTEV-A101 vector for mRNA transcription in vitro was a gift from Dr. Drew Weissman (Perelman School of Medicine, University of Pennsylvania), and it consists of T7 promoter, 5′ UTR, 3′ UTR, and poly-A tail. The synthetic DNA fragment encoding the full-length spike protein with diproline mutation of the ancestral SARS-CoV-2 strain (WHU01) was cloned into this pUC-ccTEV-A101 vector. To compare the effect of UTRs from Moderna and Pfizer-BioNTech mRNA vaccine on mRNA translation, the UTRs of pUC-ccTEV-A101 were replaced by the UTRs from Moderna (named pUC-5’M3’M), Pfizer-BioNTech (named pUC-5′P3′P), or their combination (pUC-5′M3′P and pUC-5′P3′M), and the firefly luciferase gene, as a reporter, was inserted into these vectors. In addition, in order to investigate the effect of m1Ψ content, three synthetic DNA fragments encoding the same RBD of SARS-CoV-2 spike (B.1.351) fused to a 10-mer ferritin but containing different amount of thymine (T) at the wobble position were subcloned into the pUC-ccTEV-A101 vector.

### mRNA synthesis and purification in vitro

mRNAs were transcribed from linearized plasmids encoding firefly luciferase, or full-length spike, or RBD of SARS-CoV-2 spike by the MEGAscript® T7 Transcription Kit (Cat# AMB1334-5, Life Technologies) through a modified protocol. Briefly, in order to reduce the mRNA immunity and increase its stability in vivo as COVID-19 mRNA vaccine did^1,2^, the uridine-5′-triphosphate (UTP) from the T7 Transcription Kit was replaced by m1Ψ- 5′-triphosphate (Cat# N-1081, TriLink). Meanwhile, mRNA was also capped co-transcriptionally in vitro with the trinucleotide cap1 analog CleanCap (Cat# N-7413, TriLink). After 6 h of reaction, mRNAs were precipitated by lithium chloride (LiCl) and washed twice with 75% ethanol in nuclease and endotoxin free water (Cat# W3440, Teknova). Then, the mRNA pellet was dissolved in nuclease and endotoxin free water and further subjected to cellulose purification in the presence of 16% ethanol to remove dsRNA^74^. The concentration and purity of mRNAs was measured by nanodrop and by agarose gel electrophoresis, respectively. mRNAs were aliquoted and stored at −20 °C.

### mRNA-LNP production

mRNAs were encapsulated in lipid nanoparticles by NanoAssemblr Ignite microfluidic cartridge technology (Precision Nanosystems). In brief, lipids were dissolved in ethanol at a molar ratio of 50:10:38.5:1.5 (ionizable lipid:DSPC:cholesterol:PEG2000 PE) and mRNA was prepared in 25 mM sodium acetate buffer (pH5.0) at 0.1 mg/ml. Then the lipid mixture and mRNA were mixed at an N/P ratio of 4 through the “Y” shape microfluidic cartridge at a total flow rate of 6 ml/min and a flow rate ratio of 3:1 (aqueous phase:organic phase). After formulation, the mRNA-LNP was dialyzed three times against sterile and Ca2 + /Mg^2+^ free PBS (Cat# 17-516F, Lonza) through a 3.5 K MWCO Slide-A-Lyzer dialysis cassette (Cat# 66330, Thermo Scientific), followed by concentration through a centrifugal filter YM30 (Cat# MRCF0R030, EMD Millipore). The mRNA-LNP was adjusted to 50 μg/ml with or without 10% sucrose (w/v) and aliquoted for different storage conditions. At least two independent preparations for each mRNA-LNP type were used in all experiments.

### mRNA-LNP characterization

The mRNA encapsulation efficiency and concentration were determined by Quant-iT^TM^ RiboGreen assay (Cat# R11490, Life Technologies). Briefly, mRNA-LNP samples were first diluted with 1X TE buffer (pH8.0) in the absence or presence of 1% Triton-X-100 in a black 96-well plate. Meanwhile, 20 μg/ml standard mRNA with the same lipid composition and N/P ratio was also prepared in PBS and then serially diluted with 1× TE buffer in presence of 1% Triton-X-100 to generate a standard curve. Then the plate was transferred to the 37 °C for 10 min incubation followed by addition of 100 μl 1× Ribogreen dye to each well to bind RNA. Fluorescence was measured by SpectraMax Paradigm Multi-Mode Detection Platform (Molecular Devices) at 485 nm excitation and 528 nm emission. mRNA encapsulation efficiency was calculated by the following formula: encapsulation efficiency (%) = [(Fluorescence)_total mRNA_–(Fluorescence)_outside mRNA_]/(Fluorescence)_total mRNA_ × 100%. The diameter and size distribution of the mRNA-LNP was measured by Dynamic Lighter Scattering (DLS) (DynaPro NanoStar, Wyatt Technology). After a brief vortexing, 8 μl of 50 μg/ml mRNA-LNP in PBS with or without 10% sucrose was loaded into a cuvette for a measurement at 25 °C. LNPs in PBS were read using the solvent parameter ‘water’ and those in PBS + 10% sucrose using ‘10% sucrose’. One measurement consists of four readings and each reading derives from 10 acquisitions. The DLS data presented for each mRNA-LNP preparation is the average value of these four readings.

### Administration of mRNA-LNP to mice

7-week-old female BALB/c mice from The Jackson Laboratory were used in this study. For mRNA-LNP delivery in vivo, anesthetized mice were injected with 1 μg of Fluc mRNA-LNP in 20 μl volume in the gastrocnemius muscle with a 31-gauge insulin syringe of a 0.3 ml size. Mice were anesthetized with 1–4% isoflurane in oxygen for five minutes before immunization. For experiments involving full-length spike or RBD, anesthetized mice were primed and boosted with 1 μg of mRNA-LNP vaccine on day 1 and day 21, respectively, in the same manner. The mice used in the imaging experiments were euthanized immediately after imaging, and those used in the antibody production studies were euthanized following the last bleeding on d35 post vaccination in a CO2 euthanasia chamber.

### Bioluminescence imaging and analysis

After 24 h of Fluc mRNA-LNP injection, mice were anesthetized with isoflurane and injected intraperitoneally with 120 μl of RediJect D-Luciferin Bioluminescent Substrate (Cat# 770504, PerkinElmer) and immediately placed on the warm imaging platform while being supplied with 2% isoflurane via a nose cone. Bioluminescence was measured on Lago-X (Spectral Instruments Imaging) using a group acquire setting. Bioluminescence values were quantified by measuring photon flux (photon/second) in the region of interest using Aura imaging software. As the peak bioluminescence signal might be detected at different time points, owing to the variation in metabolic status of individual mice, for data analysis, we picked from all the images of each mouse only the highest bioluminescence values within the saturation limit.

### SARS-CoV-2 pseudovirus production

SARS-CoV-2 pseudovirus (PV) entry assay was used to determine the titer of neutralizing antibodies in the plasma from the mice immunized with full-length spike or RBD mRNA-LNP. SARS-CoV-2 PV was produced as follows. Briefly, HEK293T cells at 50–60% confluency in T75 flask were transfected with 22 μg of total DNA at a ratio of 5:5:1 by mass of the retroviral vector pQCXIX expressing firefly luciferase, the plasmid encoding MLV gag and pol proteins, and the plasmid expressing dCT19-spike of SARS-CoV-2 (either WHU01 or B.1.351 strain). At 6 h post transfection, cells were washed once with PBS and replenished with 10 ml growth media. PV-containing culture supernatants were harvested at 43 h post transfection followed by clarification through 0.45 μm filters, and immediately aliquoted for storage at −80 °C.

### Plasma collection and neutralization assay

On day 14 and 35 post primary immunization, mice anesthetized with isoflurane were bled via the retro-orbital route in heparinized micro-hematocrit capillary tubes. Plasmas were collected by spinning the blood at 1500 g for 15 min at 4 °C. Individual plasma (*n* = 10 per group) was heat-inactivated at 56 °C for 30 min and serially diluted in 50 μl RPMI media containing 1 μg/ml puromycin and 10% FBS. Then 5 × 10^7 genome copies of PV expressing Fluc in 50 μl RPMI 1640 media were preincubated with the serially diluted plasmas at RT for 30 min. Preincubated samples were transferred onto ~60% confluent H1299-hACE2 cells on 96-well plates. Neutralizing ability of plasmas were assessed 24 h later by measuring firefly luciferase activity using the Luc-Pair Firefly Luciferase HS Assay Kit (Cat# LF009, GeneCopoeia). Neut_50_ was calculated through log_10_ transformation of the plasma dilution factors, at which 50% of neutralization was obtained, using default settings for log(inhibitor) vs. response variable slope method (four-parameter model) in GraphPad Prism version 9.0 (GraphPad Software lnc.).

### Inflammation response detection

In order to compare the inflammation response of mice to ALC-0315, SM-102, or cKK-E12 mRNA-LNP, the plasmas collected at 24 h post mRNA-LNP injection were analyzed for cytokines/chemokines using the Cytometric Bead Array Mouse Inflammation kit (Cat# 552364, BD Biosciences) and read by Accuri flow cytometer.

### mRNA-LNP stability test

The aliquoted Fluc mRNA-LNP (50 μg/ml) with or without 10% sucrose was removed from storage at the indicated time points (e.g. weeks 0, 4, 8, 12, and 20) and kept on ice before performing experiments. For each time point, all experiments including the measurement of particle size and encapsulation efficiency as well as mouse injection were performed on the same day. In addition, the same materials, procedures, and settings were applied to all experiments at all time points throughout the mRNA-LNP stability study. To understand whether the encapsulated mRNA is degraded after long-term storage of mRNA-LNP at different conditions, the mRNA-LNPs stored for 20 weeks were lysed with 1% Triton-X-100 in PBS for 10 min at 37 °C followed by the addition of 1× RNA loading dye (Cat# AM8552, Life Technologies) and ethidium bromide for boiling at 70 °C for 10 min. Then 200 ng of mRNA-LNP was loaded for electrophoresis to an agarose gel containing 6% formaldehyde and run in 1× MOPS buffer at 90 V for 1.5 h.

### Statistical analysis

All data were analyzed with GraphPad Prism version 9.0 (GraphPad Software Inc.) and expressed as Mean ± standard error of the mean (SEM). The difference between groups was tested using either one-way or two-way analysis of variance (ANOVA). Specific statistical analysis methods are described in the figure legends where results are presented. Values were considered statistically significant for *p* < 0.05.

### Reagent availability

Plasmids used in this study are available from the corresponding author upon request.

### Reporting summary

Further information on research design is available in the Nature Research Reporting Summary linked to this article.

### Supplementary information

Supplementary Figures

REPORTING SUMMARY

## Data Availability

All data needed to evaluate the conclusions in the paper are present in the main text and Supplementary Materials or available from the authors.
