# Supplementary material for: Effect of mRNA-LNP components of two globally-marketed COVID-19 vaccines on efficacy and stability
Source: NPJ Vaccines. 2023 Oct 11;8:156. doi: 10.1038/s41541-023-00751-6 (PMC10567765; doi:10.1038/s41541-023-00751-6)
Supplement: Supplementary file 1 — Supplementary Figures [file 41541_2023_751_MOESM1_ESM.pdf]

Supplementary Materials

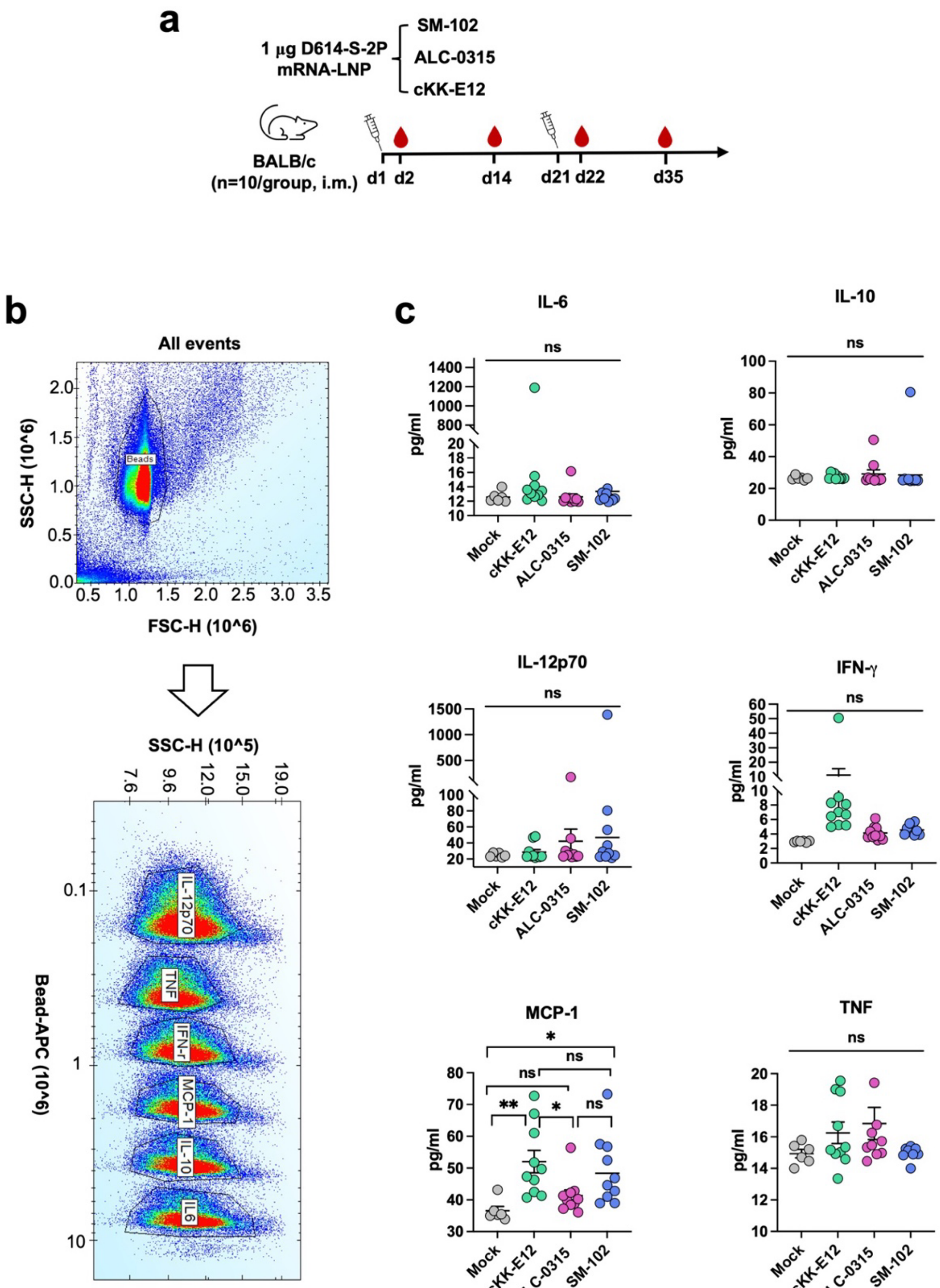

Supplementary Figure 1. (Related to Fig. 2). SM-102 and ALC-0315 LNP inflammatory responses compared to cKK-E12 LNP one day after a boost immunization. a Timeline of mouse immunization and bleeding. Ten BALB/c mice per group were intramuscularly vaccinated at the indicated days, each time with 1 μg SARS-CoV-2 spike mRNA-LNP prepared with the indicated ionizable lipid. Blood was collected at the indicated time points to measure cytokines and neutralizing antibodies. **b** Gating strategy of the flowcytometry for both d2 and d22 cytokines/chemokines detection. Within the cytokines/chemokines population, PE fluorescence intensity was measured to reflect the amount of bound cytokines/chemokines**. c** Pro-inflammatory cytokines/chemokines in the d22 plasma were measured using the murine inflammation kit and analyzed by Accuri Flow cytometer. Data are presented as Mean ± SEM of n = 10 mice per group. Statistical significance among the groups was analyzed by one-way ANOVA with Tukey's multiple comparisons test (ns, not significant; $*p < 0.05$, $**p < 0.01$).

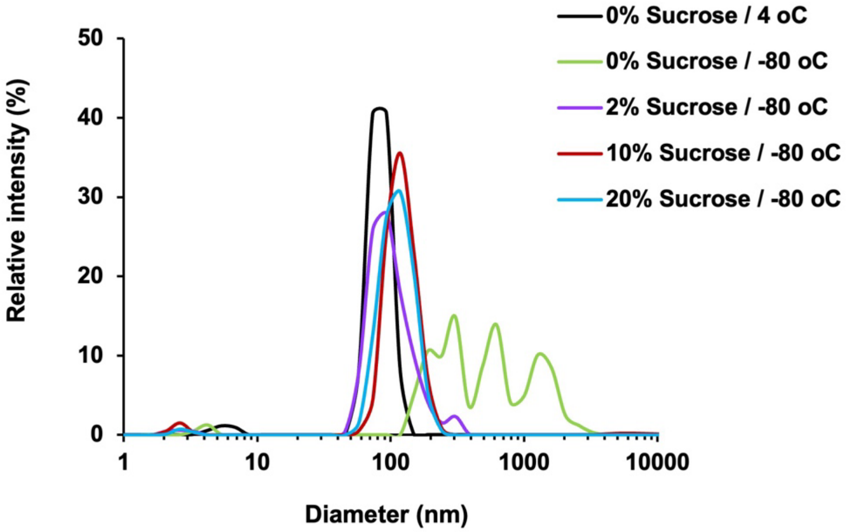

Supplementary Figure 2. (Related to Fig. 3). Effect of sucrose on mRNA-LNP size and storage at -80 °C. Size distribution of LNP was measured by DLS; mRNA-LNP was supplied with varying amount of sucrose and kept either at 4 °C or at -80 °C. LNP size was measured after thawing for those frozen at -80 °C. Sucrose protects LNP from aggregation during freeze-and-thawing. Sucrose effect on LNP size increase is observed only between 2 and 10%.

a

Pfizer/BioNTech 5'UTR:
GAGAATAAACTAGTATTCTTCTGGTCCCCACAGACTCAGAGAGAACCCGCCACC

Pfizer/BioNTech 3'UTR:
CTCGAGCTGGTACTGCATGCACGCAATGCTAGCTGCCCCTTTCCCGTCCTGGGTACCCCGAGTCTCCCCCGACC
TCGGGTCCCAGGTATGCTCCCACCTCCACCTGCCCCACTCACCACCTCTGCTAGTTCCAGACACCTCCCAAGCA
CGCAGCAATGCAGCTCAAAACGCTTAGCCTAGCCACACCCCCACGGGAAACAGCAGTGATTAACCTTTAGCAATA
AACGAAAGTTTAACTAAGCTATACTAACCCCAGGGTTGGTCAATTTCGTGCCAGCCACACCCTGGAGCTAGC

Moderna 5'UTR:
GGGAAATAAGAGAGAAAAGAAGAGTAAGAAGAAATATAAGA*CCCCGGCGCC*GCCACC

Moderna 3'UTR:
GCTGGAGCCTCGGTGGCCTAGCTTCTTGCCCCTTGGGCCTCCCCCCAGCCCCTCCTCCCCTTCCTGCACCCGTA
CCCCCGTGGTCTTTGAATAAAGTCTGAGTGGGCGGCA

TEV 5'UTR:
TCTCAACACAACATATACAAAACAAACGAATCTCAAGCAATCAAGCATTCTACTTCTATTGCAGCAATTTAAATCAT
TTCTTTTAAAGCAAAAGCAATTTTCTGAAAATTTTCACCATTTACGAACGATAGC

TEV 3'UTR:
AGTGACTGACTAGGATCTGGTTACCACTAAACCAGCCTCAAGAACACCCGAATGGAGTCTCTAAGCTACATAATA
CCAACTTACACTTACAAAATGTTGTCCCCCAAAATGTAGCCATTCGTATCTGCTCCTAATAAAAAGAAAGTTTCTTC
ACATTCT

b

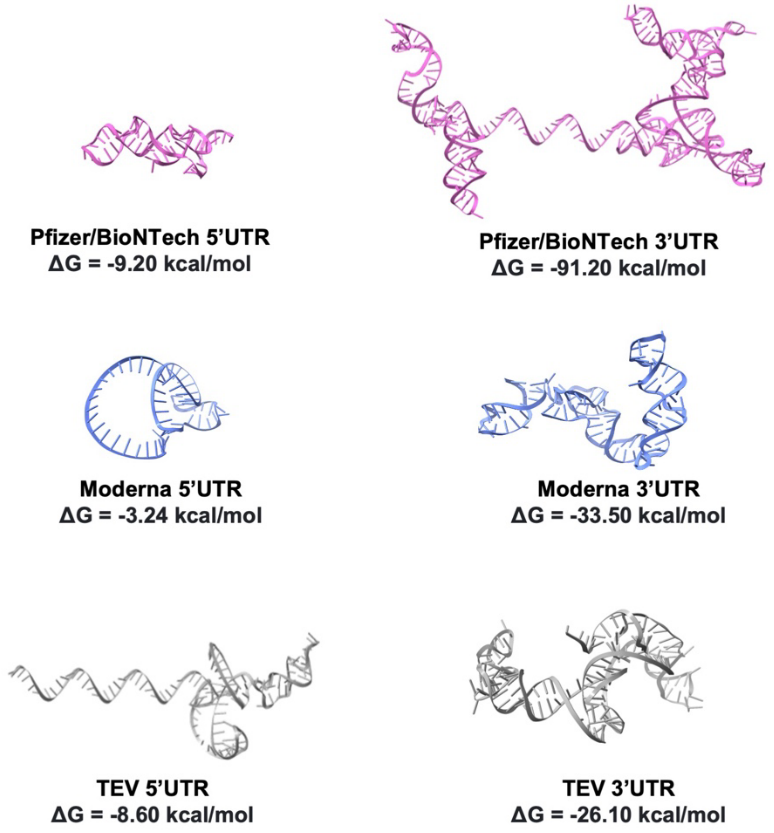

Supplementary Figure 3. (Related to Fig. 5) The sequences and secondary structures of the UTRs from the Pfizer/BioNTech and Moderna COVID-19 vaccines. a 5' and 3' UTR sequences. TEV is our own vector for *in vitro* transcription. The Kozak sequence is underlined and the high GC tract immediately upstream of the Kozak sequence in Moderna's 5' UTR is Italicized. **b** RNA secondary structures were predicted by M-fold (*http://www.unafold.org/mfold/applications/rna-folding-form.php)*, and their 3D structures were modelled using RNA Composer (*https://rnacomposer.cs.put.poznan.pl/).*

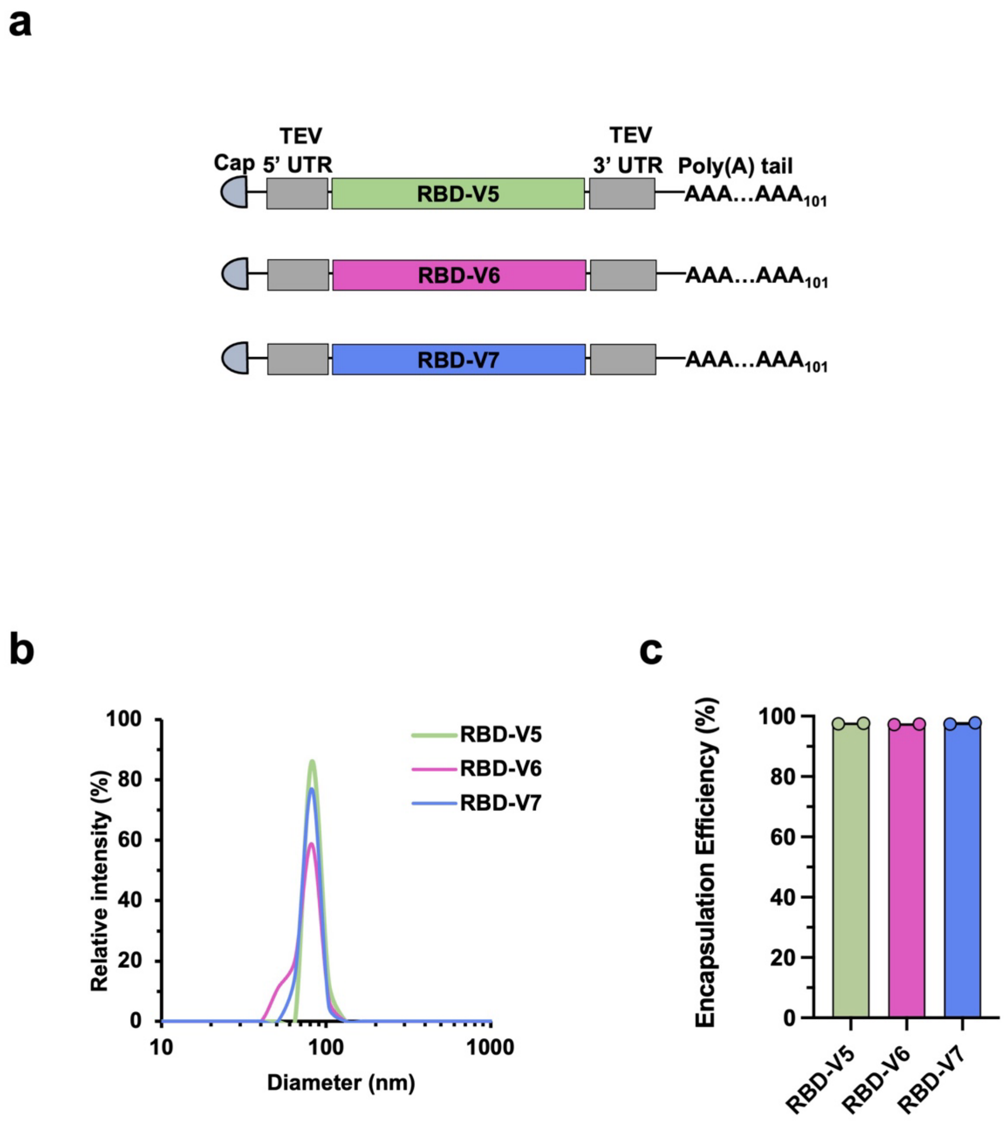

Supplementary Figure 4. (Related to Fig. 6) Particle size and encapsulation efficiency of RBD mRNA-LNPs with varying m1Ψ content. a Schematic diagrams of the RBD mRNAs used in the study with varying wobble m1Ψ content but with the same 5’ cap, UTRs, and poly(A) tail. **b** Size distribution of RBD mRNA-LNPs in 10% sucrose was measured by DLS. **c** Encapsulation efficiency of these LNPs was measured by the Quant-it™ RiboGreen RNA Assay Kit.
