# Supplementary material for: Effect of mRNA-LNP components of two globally-marketed COVID-19 vaccines on efficacy and stability
Source: NPJ Vaccines. 2023 Oct 11;8:156. doi: 10.1038/s41541-023-00751-6 (PMC10567765; doi:10.1038/s41541-023-00751-6)
Supplement: Supplementary file 2 — REPORTING SUMMARY [file 41541_2023_751_MOESM2_ESM.pdf]

nature portfolio

Corresponding author(s): Lizhou Zhang & Hyeryun Choe (Maunscript ID: NPJVACCINES-03283R)

Last updated by author(s): Sep 18, 2023

Reporting Summary

Nature Portfolio wishes to improve the reproducibility of the work that we publish. This form provides structure for consistency and transparency in reporting. For further information on Nature Portfolio policies, see our Editorial Policies and the Editorial Policy Checklist.

Statistics

For all statistical analyses, confirm that the following items are present in the figure legend, table legend, main text, or Methods section.

n/a	Confirmed	
☐	☒	The exact sample size (*n*) for each experimental group/condition, given as a discrete number and unit of measurement
☐	☒	A statement on whether measurements were taken from distinct samples or whether the same sample was measured repeatedly
☐	☒	The statistical test(s) used AND whether they are one- or two-sided<br>*Only common tests should be described solely by name; describe more complex techniques in the Methods section.*
☒	☐	A description of all covariates tested
☐	☒	A description of any assumptions or corrections, such as tests of normality and adjustment for multiple comparisons
☐	☒	A full description of the statistical parameters including central tendency (e.g. means) or other basic estimates (e.g. regression coefficient) AND variation (e.g. standard deviation) or associated estimates of uncertainty (e.g. confidence intervals)
☐	☒	For null hypothesis testing, the test statistic (e.g. *F*, *t*, *r*) with confidence intervals, effect sizes, degrees of freedom and *P* value noted<br>*Give P values as exact values whenever suitable.*
☒	☐	For Bayesian analysis, information on the choice of priors and Markov chain Monte Carlo settings
☒	☐	For hierarchical and complex designs, identification of the appropriate level for tests and full reporting of outcomes
☒	☐	Estimates of effect sizes (e.g. Cohen's *d*, Pearson's *r*), indicating how they were calculated

Our web collection on statistics for biologists contains articles on many of the points above.

Software and code

Policy information about availability of computer code

Data collection	Flow cytometry data were collected with Accuri C6 software 1.0.264 (BD) and ForeCyt 6.2R3 (IntelliCyt). Bioluminescence assay data were collected with Lago-X (Spectral Instruments Imaging) in vivo or SoftMax Pro 6.3 (Molecular Devices) in vitro. DLS data were collected with DynaPro NanoStar (Wyatt Technology). RNA gel image were collected with Image Lab 6.1 (Bio-Rad).
Data analysis	Experimental data were analyzed with GraphPad Prism version 9.0 and Microsoft Excel. DLS data were analyzed with DYNAMICS (Wyatt Technology). Mouse bioluminescent images were analyzed with Aura imaging software (Spectral Instruments Imaging).

For manuscripts utilizing custom algorithms or software that are central to the research but not yet described in published literature, software must be made available to editors and reviewers. We strongly encourage code deposition in a community repository (e.g. GitHub). See the Nature Portfolio guidelines for submitting code & software for further information.

Data

Policy information about availability of data

All manuscripts must include a data availability statement. This statement should provide the following information, where applicable:

- Accession codes, unique identifiers, or web links for publicly available datasets
- A description of any restrictions on data availability
- For clinical datasets or third party data, please ensure that the statement adheres to our policy

All data needed to evaluate the conclusions in the paper are present in the paper and/or the Supplementary Materials.

Research involving human participants, their data, or biological material

Policy information about studies with human participants or human data. See also policy information about sex, gender (identity/presentation), and sexual orientation and race, ethnicity and racism.

Reporting on sex and gender	This study does not involve the human participants.
Reporting on race, ethnicity, or other socially relevant groupings	This study does not involve the human participants.
Population characteristics	This study does not involve the human participants.
Recruitment	This study does not involve the human participants.
Ethics oversight	This study does not involve the human participants.

Note that full information on the approval of the study protocol must also be provided in the manuscript.

Field-specific reporting

Please select the one below that is the best fit for your research. If you are not sure, read the appropriate sections before making your selection.

☒ Life sciences ☐ Behavioural & social sciences ☐ Ecological, evolutionary & environmental sciences

For a reference copy of the document with all sections, see nature.com/documents/nr-reporting-summary-flat.pdf

Life sciences study design

All studies must disclose on these points even when the disclosure is negative.

Sample size	The sample size was 5-10 mice per group, and at least two independent mRNA-LNP preps were used,
Data exclusions	No data was excluded from analyses.
Replication	All mouse experiments were replicated with at least two independent mRNA-LNP preps. All other experiments including DLS, encapsulation efficiency of the mRNA-LNP, and neutralization assays were performed twice independently with at least two technical replicates.
Randomization	All the mice purchased and received from the vendor were pooled and then randomly allocated into different groups to test mRNA-LNP delivery efficiency or antibody production.
Blinding	Blinding was not performed because this is not a case-control study. The authors could not be blinded during the procedures. In addition, blinding is not necessary because this study is exclusively based on quantitative measurements using in vitro experiments.

Reporting for specific materials, systems and methods

We require information from authors about some types of materials, experimental systems and methods used in many studies. Here, indicate whether each material, system or method listed is relevant to your study. If you are not sure if a list item applies to your research, read the appropriate section before selecting a response.

Materials & experimental systems

n/a	Involved in the study	
☒	☐	Antibodies
☐	☒	Eukaryotic cell lines
☒	☐	Palaeontology and archaeology
☐	☒	Animals and other organisms
☒	☐	Clinical data
☒	☐	Dual use research of concern
☒	☐	Plants

Methods

n/a	Involved in the study	
☒	☐	ChIP-seq
☐	☒	Flow cytometry
☒	☐	MRI-based neuroimaging

Eukaryotic cell lines

Policy information about cell lines and Sex and Gender in Research

Cell line source(s)	HEK293T (Cat # CRL-3216) was purchased from ATCC. NCI-H1299 (ATCC CRL-5803D) cell line was obtained from Joseph Kissil (Scripps Research, FL). And H1299-hACE2 stable cell line was made by transducing NCl-H1299 with MLV pseduovirus expressing hACE2.
Authentication	HEK293T was not separately authenticated because they were purchased from ATCC with certificates. NCIH1299 (FTA Barcode #: STRB3886) was authenticated by ATCC Cell Line Authentication Service, using Short Tandem Repeat (STR) analysis as described in 2012 in ANS Standard (ASN-0002) Authentication of Human Cell Lines: Standardization of STR Profiling by the ATCC Standards Development Organization (SDO) and in Capes-Davis et al., Match criteria for human cell line authentication: Where do we draw the line? Int. J. Cancer. 2012 Nov 8. doi:10.1002/ijc.27931
Mycoplasma contamination	Cells were checked for the presence of mycoplasma before freezing them, using two different methods: DAPI staining for the presence of mycoplasma DNA and PlasmoTest (InVivogen) that detects all mycoplasmas through TLR2-mediated recognition. Thawed cells were not tested for mycoplasma , because they were tested negative before frozen, but maintained in the presence of prophylactic concentration (2.5 mg/ml) of Plasmocin antimycotic (InVivoGen). Antimycotic is used only for maintenance, and cells used in experiments are plated in media lacking antimycotic.
Commonly misidentified lines (See ICLAC register)	No commonly misidentified cell lines were used.

Animals and other research organisms

Policy information about studies involving animals; ARRIVE guidelines recommended for reporting animal research, and Sex and Gender in Research

Laboratory animals	Mouse, BALB/c, 7-8 weeks
Wild animals	This study did not use wild animals.
Reporting on sex	This study only used female BALB/c mice because female mice produce higher level of antibodies in response to a vaccine based on the previous study (https://doi.org/10.1016/S0145-305X(81)80040-7).
Field-collected samples	This study did not include samples collected from the field.
Ethics oversight	All study procedures were approved by the Institutional Biosafety Committee, and Institutional Animal Care and Use Committee at The UF Scripps Institute for Biomedical Innovation & Technology. All experiments conform to all relevant regulatory standards.

Note that full information on the approval of the study protocol must also be provided in the manuscript.

Flow Cytometry

Plots

Confirm that:

- [x] The axis labels state the marker and fluorochrome used (e.g. CD4-FITC).
- [x] The axis scales are clearly visible. Include numbers along axes only for bottom left plot of group (a 'group' is an analysis of identical markers).
- [x] All plots are contour plots with outliers or pseudocolor plots.
- [ ] A numerical value for number of cells or percentage (with statistics) is provided.

Methodology

Sample preparation	Mouse plasmas collected at 24 hours post mRNA-LNP injection were diluted and then mixed with cytokine capture beads followed by incubation with PE conjugated detection antibodies.
Instrument	Accuri C6
Software	ForeCyt 6.2R3 (IntelliCyt)
Cell population abundance	There were six kind of beads coated with capture antibodies specific for IL-6, IL-10, MCP-1, IFN-r, TNF, and IL-12p70, which emit distinct intensity of fluorescence.

Gating strategy

The major bead population was first gated in the FSC/SSC plot, then the cytokine specific populations were further gated by the distinct APC intensities according to the manufacturer's manual. Within each cytokine population, PE fluorescence intensity was measured to assess the amount of bound cytokines .

☒ Tick this box to confirm that a figure exemplifying the gating strategy is provided in the Supplementary Information.
